# Doppler-Guided Haemorrhoidal Artery Ligation and Rectoanal Repair (HAL-RAR): An Institutional Experience

**DOI:** 10.3390/jcm14155397

**Published:** 2025-07-31

**Authors:** Rathin Gosavi, Raelene Tan, David Zula, Simon Xu, Shiki Fujino, James Lim, Thang Chien Nguyen, William Teoh, Vignesh Narasimhan

**Affiliations:** 1 Department of Colorectal Surgery, Cabrini Health, Melbourne, VIC 3144, Australia; 2 Department of Colorectal Surgery, Monash Health, Melbourne, VIC 3175, Australia; 3 Department of Surgery, School of Clinical Sciences at Monash Health, Monash University, Melbourne, VIC 3168, Australia

**Keywords:** Haemorrhoids, HAL-RAR, DG-HALRAR

## Abstract

**Background**: Doppler-guided haemorrhoidal artery ligation with rectoanal repair (HAL-RAR) is a minimally invasive alternative to conventional haemorrhoidectomy. While associated with reduced postoperative pain and quicker recovery, data on its safety, recurrence rates, and applicability across haemorrhoid grades remain limited, particularly in Australian settings. **Methods**: A retrospective review was conducted on 128 consecutive patients who underwent elective HAL-RAR at a single institution between February 2022 and December 2024. Data on demographics, operative details, postoperative outcomes, and recurrence were collected. Outcomes were stratified by haemorrhoid grade. Multivariate logistic regression was used to identify predictors of recurrence, day-case completion, and conversion to excisional surgery. **Results:** The median age was 49 years, and 77.3% had Grade II or III haemorrhoids. HAL-RAR was completed as a day case in 76.6% of patients. Postoperative urinary retention occurred in 3.9%, return to theatre in 0.8%, and 30-day readmission in 7.0%. The symptomatic recurrence rate was 17.6%. Grade IV haemorrhoids were independently associated with increased recurrence (aOR 3.64, 95% CI 1.03–12.84), reduced likelihood of day-case management (aOR 0.14, 95% CI 0.03–0.93), and higher conversion to excisional haemorrhoidectomy (aOR 7.23, 95% CI 1.13–46.40). **Conclusions:** HAL-RAR is a safe, effective, and low-morbidity option for the management of Grade II and III haemorrhoids, suitable for day-case surgery. In selected Grade IV cases, it may offer benefit, although with higher recurrence and conversion risk. Careful patient selection is essential, and longer-term prospective studies are needed to assess durability.

## 1. Introduction

Haemorrhoidal disease is a common anorectal condition characterised by bleeding, discomfort, pruritus, and mucosal prolapse, often impairing quality of life and daily function [1]. While Grade I and some Grade II haemorrhoids can usually be managed conservatively or with rubber band ligation/sclerotherapy, Grade III–IV disease traditionally requires excisional haemorrhoidectomy most cases, as described by Milligan–Morgan [2] and Ferguson [3]. While very effective, excisional techniques are associated with considerable postoperative pain, prolonged recovery, and procedure-related morbidity [4]. Up to 65% of patients report moderate to severe pain beyond the first postoperative week, and recovery commonly requires 2–3 weeks off work [5]. Postoperative complications such as urinary retention, bleeding, and delayed wound healing occur in up to 20% of cases, contributing to patient dissatisfaction and healthcare burden [6].

In response to these limitations, non-excisional alternatives have emerged, including Doppler-guided haemorrhoidal artery ligation with rectoanal repair (HAL-RAR) [7,8,9]. Initially described by Maringa et al. [10] and later refined to include mucopexy, HAL-RAR targets the underlying vascular and mechanical contributors to haemorrhoidal disease and offers a less morbid alternative to haemorrhoidectomy in patients with more advanced haemorrhoids. Doppler-guided ligation reduces arterial inflow to the haemorrhoidal plexus, leading to shrinkage of the vascular cushions, while the rectoanal repair component mechanically lifts and secures prolapsed mucosa back into its anatomical position through a running mucopexy. This dual approach alleviates symptoms and reduces the risk of recurrent prolapse without tissue excision [9]. Compared with excisional approaches, HAL-RAR is associated with reduced pain, lower complication rates, and earlier return to normal activities [11].

Despite uptake internationally, outcomes data from Australasian centres remain limited. Since 2022, our institution has adopted HAL-RAR for patients with Grade II/III haemorrhoids and select Grade IV cases. This study aims to evaluate the safety, short-term efficacy, and recurrence rates associated with HAL-RAR as a treatment option for haemorrhoids. Secondary outcomes include length of stay and perioperative complication rates.

## 2. Methods

A retrospective cohort study was conducted on consecutive patients who underwent a Doppler-guided HAL-RAR at Monash Health between 2 February 2022 and 2 December 2024.

### 2.1. Inclusion Criteria

All patients over the age of 18 who underwent elective Doppler-guided HAL-RAR, either for primary presentation or for recurrent haemorrhoidal disease following previous interventions, were eligible for inclusion. Patients with a history of rubber band ligation, phenol injection, or prior excisional haemorrhoidectomy were included, provided HAL-RAR was performed electively and not as a salvage procedure in the setting of acute complications. At our institution, both HAL-RAR and conventional excisional haemorrhoidectomy are routinely performed. The choice of procedure is based on haemorrhoid grade, symptom severity, anatomical features, and patient preference. HAL-RAR is typically offered for Grade II and III disease, and selectively for Grade IV cases where the prolapse is predominantly internal with minimal or no external component. Patients with a bulky external component or circumferential prolapse are generally offered excisional surgery due to the reduced likelihood of success with non-excisional techniques.

### 2.2. Follow-Up and Recurrence

Patients were routinely reviewed in the outpatient clinic at approximately 4 to 6 weeks postoperatively. Those with complete symptom resolution at this initial review were discharged from further follow-up. Patients were advised to reattend if symptoms recurred and were eligible for re-referral or direct rebooking within a 12-month period.

Recurrence was defined as the reappearance of haemorrhoidal symptoms (e.g., prolapse or bleeding) after initial symptom resolution and discharge, prompting clinical reassessment or re-intervention. Recurrence events were identified through scheduled follow-up or unplanned presentations recorded in the electronic medical record.

As a public institution with a defined referral catchment, most patients were expected to re-present to our service for recurrence. However, we acknowledge that some individuals may have sought care elsewhere. No formal cross-institutional data linkage was available, and the recorded recurrence rate reflects only those captured within our electronic medical records.

### 2.3. Ethics Approval

This study was approved as a low-risk project by the Monash Health Human Research Ethics Committee (RES-25-0000-112Q), with a waiver of individual patient consent due to its retrospective design.

### 2.4. Surgical Technique

The surgical technique was performed in a standardised manner by all members in the unit. All procedures were performed under general anaesthesia in the lithotomy position. Patients all received a fleet enema preoperatively. A single dose of intravenous metronidazole 500 mg was administered. Bilateral pudendal blocks were administered, following which an examination under anaesthesia (EUA) was conducted and the HAL-RAR platform was docked, paired to the speaker via Bluetooth so arterial wave signals were audibly transmitted to assist with vessel localisation. The HAL-RAR procedure was performed using the A.M.I. HAL-Doppler II proctoscope system (A.M.I. Ltd, Feldkirch, Austria). A 20 MHz Doppler probe was used to identify terminal branches of the superior rectal artery, which were ligated using 2/0 absorbable sutures (Vicryl^®^) in a figure-of-eight configuration. The number of ligations performed in each case varied, depending on Doppler mapping. Following the haemorrhoidal artery ligation (HAL), rectoanal repair (RAR) was performed using the same continuous 2.0 Vicryl suture to plicate the mucosa distally, with the terminal sutures placed above the dentate line. The depth and number of sutures were determined by the extent of mucosal laxity and degree of prolapse. Suture placement avoided the muscularis and was adjusted to achieve symmetrical mucosal lift and restoration of the anorectal contour. Postoperatively, patients were started on Movicol daily and given simple analgesia in the form of Paracetamol and non-steroidals (NSAIDs). Tapendalol was prescribed to be used on an as-needed basis. Patients were discharged home when pain control was adequate and had no evidence of urinary retention.

### 2.5. Data Collection

Preoperative, intraoperative, and postoperative data were extracted from electronic medical records. Collected variables included demographic details (age, sex, body mass index (BMI)), comorbidity status (American Society of Anaesthesiologists (ASA) score), presenting symptoms (bleeding, prolapse, or both), haemorrhoidal grade, prior treatments (rubber band ligation, sclerotherapy, excisional haemorrhoidectomy), number of pedicles ligated, intraoperative complications, length of stay, postoperative complications (urinary retention, readmission, return to theatre), and follow-up duration. Recurrence was defined as the return of haemorrhoidal symptoms (bleeding or prolapse) following initial resolution and was identified through clinician assessment during scheduled or symptom-prompted follow-up visits.

### 2.6. Statistical Analysis

Continuous variables were reported as medians with range, and categorical variables as frequencies and percentages. Univariate and multivariate logistic regression analyses were performed to identify predictors of recurrence, 30-day readmission, and postoperative complications. Covariates included haemorrhoid grade (used as the definitive classification), BMI, ASA score, number of pedicles ligated, and history of prior interventions.

All statistical analyses were conducted using STATA version 18, with a *p*-value of <0.05 considered statistically significant.

## 3. Results

### 3.1. Baseline Demographics

A total of 128 patients were included in the study. The median age was 49 years (24–86), with over half being male (55.5%). The median BMI was 26.3 (17.6–40.1). The ASA physical status score was as follows: ASA I in 45 patients (35.2%), ASA II in 61 (47.7%), ASA III in 20 (15.6%), and ASA IV in 2 patients (1.6%). Thirty seven patients (28.9%) had previously had haemorrhoidal treatment. Most of these included rubber band ligation (RBL) (n = 33), with the other four patients having had a previous haemorrhoidectomy. Demographics of the cohort are summarised in Table 1.

### 3.2. Presentation and Indication for Surgery

The primary symptoms among the patient cohort were categorised into bleeding, prolapse, or a combination of both. The most commonly reported presenting symptom was bleeding (n = 55, 43.0%). A further 52 patients (40.6%) reported both symptoms of haemorrhoidal prolapse and bleeding, while 21 patients (16.4%) reported prolapse alone. Based on clinical history and examination prior to surgery, the haemorrhoids were classified as Grade I in 7 patients (5.5%), Grade II in 38 patients (29.7%), Grade III in 61 patients (47.7%), and Grade IV in 22 patients (17.2%).

### 3.3. Intraoperative Details

The median number of haemorrhoidal pedicles ligated was 4 (range: 1–7). In 117 patients (91.4%), the HAL-RAR procedure was completed without the need for adjunctive procedures. In 11 patients (8.6%), intraoperative conversion to excisional haemorrhoidectomy was required due to intraoperative findings such as extensive prolapse or brisk bleeding.

### 3.4. Postoperative Outcomes

A total of 98 patients (76.6%) were managed as day procedures. The remaining 30 patients (23.4%) required inpatient admission, primarily for postoperative observation or delayed recovery from anaesthesia. Among admitted patients, the median length of stay was 1 day (range 1–2).

Postoperative urinary retention occurred in five patients (3.9%). All cases were managed with insertion of an indwelling catheter (IDC) and a trial of void (TOV) the following day. No patients required ongoing catheterisation or experienced long-term sequelae. Return to theatre within 30 days occurred in only one patient (0.8%) who re-presented with persistent postoperative bleeding on day 2, which was successfully managed operatively with suture haemostasis. The patient was discharged the subsequent day with no further bleeding. The 30-day readmission rate was 7.0% (n = 9), with common causes including per-rectal bleeding (n = 4), constipation (n = 3), and pain (n = 2). No major morbidity or mortality was observed (Table 2).

### 3.5. Follow-Up and Management of Recurrence

Twenty-two patients (17.6%) were identified as having symptomatic recurrence (Table 2). The mean time to recurrence was approximately 16 months (range 11–20 months). The predominant recurrent symptom was prolapse (n = 11, 50.0%), followed by bleeding (n = 9, 40.9%), and both symptoms in two patients (9.1%). Among the 22 patients with a symptomatic recurrence, 12 patients underwent further intervention: redo HAL-RAR (n = 7), excisional haemorrhoidectomy (n = 3), and phenol injection (n = 2).

The average follow-up duration was 13.4 weeks. Eleven patients (8.6%) were lost to follow-up due to non-attendance at scheduled outpatient clinic appointments.

Only 28 patients (21.9%) required follow-up extending beyond 6 months, with 15 patients (11.7%) beyond 12 months.

### 3.6. Outcomes by Haemorrhoidal Grade

Subgroup analysis comparing patients with Grade II/III haemorrhoids to those with Grade IV disease demonstrated key differences in clinical outcomes.

Grade IV disease was independently associated with a higher risk of symptomatic recurrence following HAL-RAR, with an adjusted odds ratio (aOR) of 3.64 (95% CI 1.03–12.84, *p* = 0.04) (Table 3).

Patients with Grade IV haemorrhoids were also significantly less likely to be discharged on the same day. The adjusted odds of undergoing HAL-RAR as a day procedure were markedly lower in this group (aOR 0.14, 95% CI 0.03–0.93, *p* = 0.04) (Table 4). Additionally, intraoperative conversion to excisional haemorrhoidectomy was more common in patients with Grade IV disease, with an adjusted odds ratio of 7.23 (95% CI 1.13–46.40, *p* = 0.04) (Table 5).

## 4. Discussion

Doppler-guided haemorrhoidal artery ligation with rectoanal repair (HAL-RAR) is increasingly used as a minimally invasive alternative to excisional haemorrhoidectomy in many cases. Its theoretical advantage lies in targeting both vascular and mechanical contributors to haemorrhoidal disease. By reducing arterial inflow and repositioning prolapsed tissue, HAL-RAR seeks to alleviate symptoms while avoiding the morbidity of tissue excision [12]. These advantages have made it a popular option for patients seeking faster recovery, less pain, and earlier return to daily function.

This study reinforces the safety, effectiveness, and practical versatility of Doppler-guided HAL-RAR in managing symptomatic haemorrhoidal disease, particularly for Grade II and III presentations. The majority of patients (77%) were treated as day cases, with minimal morbidity and no major complications. Urinary retention occurred in fewer than 4% of cases, and return to theatre was rare (0.8%). Readmissions were infrequent and typically minor. These findings align with the minimally invasive intent of HAL-RAR and support its use as an efficient, well-tolerated intervention that enables rapid recovery and minimal hospital resource utilisation.

The procedure was successfully completed in 91.4% of cases without the need for conversion to excisional techniques. Among the 8.6% who required conversion, intraoperative factors such as bulky prolapse or uncontrolled bleeding were the primary drivers. This illustrates the adaptability of HAL-RAR and the importance of intraoperative decision-making.

Importantly, subgroup analysis highlighted that patients with Grade IV disease had significantly higher odds of symptomatic recurrence (aOR 3.64), lower likelihood of same-day discharge (aOR 0.14), and higher rates of intraoperative conversion to excisional haemorrhoidectomy (aOR 7.23). While HAL-RAR remains technically feasible in select Grade IV cases, these results suggest it should be offered with caution, and only when patients are appropriately counselled regarding its limitations. In contrast, patients with Grade II and III haemorrhoids experienced favourable outcomes with low recurrence and high day-case completion, reinforcing HAL-RAR as a strong first-line option in this group.

Recurrence remains a key concern in the assessment of HAL-RAR’s long-term efficacy. In this cohort, the overall recurrence rate was 17.6%, consistent with existing literature, which reports rates ranging from 10% to 30% depending on follow-up duration and patient selection [13,14]. Most recurrences were characterised by mild to moderate symptoms, primarily prolapse or bleeding. The choice of re-intervention in this series was based on the severity and nature of recurrent symptoms, as well as patient preference. Redo HAL-RAR was typically offered to patients with persistent prolapse or bleeding without extensive external components, while excisional haemorrhoidectomy was reserved for refractory or high-grade recurrence. Of those with recurrence, 54.5% (12 of 22 patients) required further treatment. Importantly, the majority of these cases were managed without excision: 31.8% underwent redo HAL-RAR, 9.1% received phenol injection, and only 13.6% required excisional haemorrhoidectomy. These findings suggest that recurrence after HAL-RAR can often be addressed with conservative or minimally invasive strategies, supporting its role in a staged treatment paradigm with flexibility for re-treatment.

Haemorrhoid grade was identified as the only statistically significant predictor of recurrence. This association remained significant after adjustment for age, sex, BMI, ASA score, presenting symptoms, number of pedicles ligated, and prior interventions. This suggests that disease severity at the time of surgery, rather than technical factors alone, plays a central role in determining long-term success. The increased risk of recurrence observed particularly in patients with Grade IV disease likely reflects more advanced mucosal prolapse or connective tissue laxity, which may not be fully corrected by HAL-RAR alone. This carries both clinical and methodological implications. First, it reinforces the prognostic value of visual assessment, which should be documented routinely. Second, it may guide postoperative counselling, especially in patients with high-grade disease who may benefit from closer follow-up or earlier consideration of excisional treatments. Additionally, consideration should be given to excisional haemorrhoidectomy for Grade IV haemorrhoids as the optimal treatment. Interestingly, other potential predictors such as BMI, ASA score, and the number of pedicles treated did not show a significant association with recurrence. This suggests that technical adequacy, while essential, may be less influential than disease severity in determining long-term success.

Most recurrences were identified at a mean of 16 months postoperatively. This likely reflects a common postoperative trajectory in which patients with initial symptom resolution are discharged early, but later re-present when symptoms recur, rather than remaining under continuous surveillance. Consequently, follow-up data disproportionately represents patients with recurrence, and the true recurrence rate among the entire cohort may be underestimated. Additionally, recurrence following HAL-RAR is likely multifactorial and influenced by patient-related variables such as bowel habit, straining, diet, and pelvic floor dysfunction. These factors were not routinely documented in this retrospective study. These findings underscore the importance of longer-term prospective follow-up and careful patient selection to more accurately evaluate procedural durability.

Comparative data across haemorrhoidal interventions reveal a spectrum of efficacy and morbidity profiles. Excisional haemorrhoidectomy, while offering the lowest recurrence rates (typically < 5%), is associated with high postoperative pain and longer recovery [15]. Stapled haemorrhoidopexy (Longo) achieves lower pain scores but has reported recurrence rates of 10–20% and risk of rare but serious complications such as rectal perforation [16]. Laser haemorrhoidoplasty offers a less invasive option with recurrence rates ranging from 10 to 25%, though robust long-term data remain limited [17]. In this context, HAL-RAR offers an intermediate profile, less painful than excisional surgery, with recurrence rates in the range of 10–30%, and the benefit of re-treatability in appropriately selected patients.

This study has several limitations. First, its retrospective design introduces inherent risks of selection and information bias. Second, the absence of a direct control group limits comparison against alternative interventions. However, identifying a suitable comparator remains challenging: excisional haemorrhoidectomy is often considered excessive for Grade II/III disease due to its associated morbidity, while HAL-RAR may be insufficient in more advanced Grade IV cases. As such, there is no universally accepted standard against which to benchmark HAL-RAR across all grades, reinforcing the need for stratified analyses and grade-specific trials. Third, the modest sample size and low event rates reduce the power to detect small effect sizes and may increase the risk of type II error in multivariate modelling. Variable follow-up duration and the 8.6% loss to follow-up introduce further risk of attrition bias, possibly underestimating recurrence. Additionally, relevant clinical variables such as anticoagulant use, laxative compliance, and bowel habits were not routinely recorded and thus excluded from analysis. These limitations highlight the need for prospective, controlled studies with longer and standardised follow-up, patient-reported outcomes, and robust cost-effectiveness data.

Future directions should include prospective studies with standardised patient-reported outcomes, longer follow-up periods, and cost-effectiveness analyses. Comparative trials with excisional techniques in higher-grade disease may further clarify HAL-RAR’s place in the therapeutic hierarchy. Until such data are available, HAL-RAR remains a valuable tool in the surgical armamentarium for haemorrhoidal disease, especially when employed judiciously in appropriately selected patients.

## 5. Conclusions

HAL-RAR is a safe and effective minimally invasive option for the treatment of symptomatic haemorrhoidal disease, particularly in patients with Grade II and III disease. It is well-suited to day-case management and is associated with low perioperative morbidity and acceptable early recurrence rates. While feasible in selected Grade IV cases, this group demonstrated higher recurrence and conversion rates, suggesting the need for careful patient selection and counselling. HAL-RAR offers a valuable alternative to excisional haemorrhoidectomy in appropriately selected patients and warrants further study to define its long-term durability and cost-effectiveness.

## Figures and Tables

**Table 1:** Demographics.

Variable	Value
Median age	49.2 (range 24–86)
Sex (M/F)	71/57
ASA < III	83.5%
ASA > III	16.4%
Any prior haemorrhoidal treatment (Yes)	47%
Presenting symptom: Bleeding only	43%
Presenting symptom: Prolapse only	15.6%
Presenting symptom: Both	40.6%
Grade II–III	82.8%
Grade IV	17.2%

**Table 2:** Postoperative outcomes and recurrence.

Postoperative Outcome	Value
Urinary retention	3.9%
Return to theatre	2.3%
30-day readmission	7.8%
Symptomatic recurrence Bleeding only Prolapse only Both	22 patients (17.6%) 40.9% 50% 9.1%
Re-intervention Redo HALRAR Phenol injection Excisional haemorrhoidectomy	12 patients (9.4%) 58.3% 16.7% 25%

**Table 3:** Haemorrhoid recurrence rate by haemorrhoid grade after HALRAR.

Variables	Total n = 119	Haemorrhoid Grades					
		Grade II/III n = 92	Grade IV n = 27	OR (95% CI)	*p*-Value	OR Adjusted * (95% CI)	*p*-Value Adjusted *
Recurrence rate of haemorrhoid (yes, %)	24 (20)	16 (17)	8 (30)	2.00 (0.74–5.36)	0.17	3.64 (1.03–12.84)	0.04

* adjusted to gender, BMI, age, ASA, presenting symptoms, previous surgeries, haemorrhoid pedicle numbers, any post-op complications, conversion rate, and pre-op haemorrhoid grades.

**Table 4:** Rate of day cases post HALRAR by haemorrhoid grade.

Variables	Total n = 128	Haemorrhoid Grades					
		Grade II/III n = 97	Grade IV n = 31	OR (95% CI)	*p*-Value	OR Adjusted * (95% CI)	*p*-Value Adjusted *
Day case (yes, %)	38 (23)	27 (29)	2 (10)	0.28 (0.08–0.99)	0.05	0.14 (0.03–0.93)	0.04

* adjusted to gender, BMI, age, ASA, presenting symptoms, previous surgeries, haemorrhoid pedicle numbers, any post-op complications, conversion rate, and pre-op haemorrhoid grades.

**Table 5:** Rate of intra-op conversion to haemorrhoidectomy by haemorrhoid grade.

**Variables**	**Total** **n = 128**	**Haemorrhoid Grades**					
		**Grade II/III** **n = 97**	**Grade IV** **n = 31**	**OR (95% CI)**	***p*** **-Value**	OR Adjusted* (95% CI)	***p*** -Value Adjusted *
Conversion to haemorrhoidectomy (yes, %)	10 (8)	6 (6)	4 (13)	2.25 (0.59–8.55)	0.24	7.23 (1.13–46.40)	0.04

* adjusted to gender, BMI, age, ASA, presenting symptoms, previous surgeries, haemorrhoid pedicle numbers, any post-op complications, and pre-op haemorrhoid grades.

## Data Availability

De-identified data supporting the findings of this study are available upon reasonable request from the corresponding author.

## References

[1] Pata F., Sgro A., Ferrara F., Vigorita V., Gallo G., Pellino G. (2021). Anatomy, Physiology and Pathophysiology of Haemorrhoids. Rev. Recent Clin. Trials.

[2] (1985). Classic articles in colonic and rectal surgery. Edward Thomas Campbell Milligan 1886–1972. Surgical anatomy of the anal canal, and the operative treatment of haemorrhoids. Dis. Colon Rectum.

[3] Ferguson J.A., Heaton J.R. (1959). Closed hemorrhoidectomy. Dis. Colon Rectum.

[4] Johannsson H.O., Pahlman L., Graf W. (2013). Functional and structural abnormalities after milligan hemorrhoidectomy: A comparison with healthy subjects. Dis. Colon Rectum.

[5] Lohsiriwat V., Jitmungngan R. (2022). Strategies to Reduce Post-Hemorrhoidectomy Pain: A Systematic Review. Medicina.

[6] Yeo D., Tan K.Y. (2014). Hemorrhoidectomy—Making sense of the surgical options. World J. Gastroenterol..

[7] Emile S.H., Elfeki H., Sakr A., Shalaby M. (2019). Transanal hemorrhoidal dearterialization (THD) versus stapled hemorrhoidopexy (SH) in treatment of internal hemorrhoids: A systematic review and meta-analysis of randomized clinical trials. Int. J. Colorectal Dis..

[8] Popov V., Yonkov A., Arabadzhieva E., Zhivkov E., Bonev S., Bulanov D., Tasev V., Korukov G., Simonova L., Kandilarov N. (2019). Doppler-guided transanal hemorrhoidal dearterilization versus conventional hemorrhoidectomy for treatment of hemorrhoids—Early and long-term postoperative results. BMC Surg..

[9] Hoyuela C., Carvajal F., Juvany M., Troyano D., Trias M., Martrat A., Ardid J., Obiols J. (2016). HAL-RAR (Doppler guided haemorrhoid artery ligation with recto-anal repair) is a safe and effective procedure for haemorrhoids. Results of a prospective study after two-years follow-up. Int. J. Surg..

[10] Morinaga K., Hasuda K., Ikeda T. (1995). A novel therapy for internal hemorrhoids: Ligation of the hemorrhoidal artery with a newly devised instrument (Moricorn) in conjunction with a Doppler flowmeter. Am. J. Gastroenterol..

[11] Ratto C., Campenni P., Papeo F., Donisi L., Litta F., Parello A. (2017). Transanal hemorrhoidal dearterialization (THD) for hemorrhoidal disease: A single-center study on 1000 consecutive cases and a review of the literature. Tech. Coloproctol..

[12] Denoya P.I., Fakhoury M., Chang K., Fakhoury J., Bergamaschi R. (2013). Dearterialization with mucopexy versus haemorrhoidectomy for grade III or IV haemorrhoids: Short-term results of a double-blind randomized controlled trial. Colorectal Dis..

[13] Symeonidis D., Spyridakis M., Zacharoulis D., Tzovaras G., Samara A.A., Valaroutsos A., Diamantis A., Tepetes K. (2022). Milligan-Morgan hemorrhoidectomy vs. hemorrhoid artery ligation and recto-anal repair: A comparative study. BMC Surg..

[14] Lauricella S., Palmisano D., Brucchi F., Agoglitta D., Fiume M., Bottero L., Faillace G. (2024). Long-term results and quality of life after stapled hemorrhoidopexy vs. Doppler-guided HAL-RAR: A propensity score matching analysis. Int. J. Colorectal Dis..

[15] Gallo G., Martellucci J., Sturiale A., Clerico G., Milito G., Marino F., Cocorullo G., Giordano P., Mistrangelo M., Trompetto M. (2020). Consensus statement of the Italian society of colorectal surgery (SICCR): Management and treatment of hemorrhoidal disease. Tech. Coloproctol..

[16] Lumb K.J., Colquhoun P.H., Malthaner R.A., Jayaraman S. (2006). Stapled versus conventional surgery for hemorrhoids. Cochrane Database Syst. Rev..

[17] Tan V.Z.Z., Peck E.W., Sivarajah S.S., Tan W.J., Ho L.M.L., Ng J.L., Chong C., Aw D., Mainza F., Foo F.J. (2022). Systematic review and meta-analysis of postoperative pain and symptoms control following laser haemorrhoidoplasty versus Milligan-Morgan haemorrhoidectomy for symptomatic haemorrhoids: A new standard. Int. J. Colorectal Dis..

